# Telehealth Use and Modality Choice Among US Adults: Shorrocks-Shapley Decomposition of a 2022 Cross-Sectional National Survey

**DOI:** 10.2196/81879

**Published:** 2026-03-18

**Authors:** Corneliu Bolbocean, Corey Hayes, Cari Bogulski

**Affiliations:** 1 Nuffield Department of Primary Care Health Sciences, University of Oxford, Radcliffe Primary Care Building, Radcliffe Observatory Quarter, Woodstock Rd, Oxford, OX2 6GG, United Kingdom, +44 1865 617855; 2 Department of Pharmacy Practice, University of Arkansas for Medical Sciences, Little Rock, AR, United States; 3 Department of Biomedical Informatics in the College of Medicine, UAMS, Little Rock, AR, United States

**Keywords:** telehealth, digital divide, eHealth literacy, Shorrocks-Shapley decomposition, Health Information National Trends Survey, HINTS

## Abstract

**Background:**

Telehealth use surged during the COVID-19 pandemic and has stabilized at levels substantially above prepandemic baselines. However, concerns persist that the digital divide may reproduce or widen disparities in access. Understanding the determinants of telehealth use—and particularly modality choice between video and audio—is essential for designing policies that promote equitable access in the post–public health emergency era.

**Objective:**

This study aims to identify determinants of telehealth use and modality among US adults in 2022 and quantify the relative contributions of digital, geographic, clinical, and socioeconomic domains.

**Methods:**

We conducted a cross-sectional secondary analysis of the sixth cycle of the Health Information National Trends Survey, administered in 2022 by the National Cancer Institute, a nationally representative, 2-stage stratified random probability survey of civilian, noninstitutionalized US adults aged 18 years or older. Sampled households were recruited via mailed invitations, and 1 adult per household was randomly selected using the next birthday method and invited to complete a self-administered questionnaire between February 2022 and November 2022 (N=6252). The primary analytic sample included respondents with nonmissing telehealth modality responses (n=6046, 59.4% female; mean age of 55.1 y). Individual-level data were linked to county-level American Community Survey socioeconomic indicators and broadband availability measures. The primary outcome was telehealth use, categorized as video (n=1641, 27.2%; 95% CI 25.5%-29.1%), audio-only (n=876, 12.1%; 95% CI 10.9%-13.4%), or none (n=3529, 60.7%; 95% CI 58.6%-62.7%). We estimated 4 binary contrasts using survey-weighted linear probability models with jackknife variance estimation, reporting absolute risk differences in percentage points (pp) with 95% CIs. We applied Shorrocks-Shapley decomposition to quantify each predictor domain’s contribution to explained variance.

**Results:**

Nationally, 39.3% (n=2517; 95% CI 37.3%‐41.4%) reported any telehealth use in the past 12 months. In survey-weighted linear probability models (*α*=.05), significant predictors of any telehealth vs none included: male sex (−9.7 pp, 95% CI −14.0 to −5.4), disability status (+22.5 pp, 95% CI 16.1-28.8), and health app use (+18.4 pp, 95% CI 12.0-24.8). For video vs audio-only telehealth, insurance coverage increased video use (+21.2 pp, 95% CI 13.0-29.3), while basic cell phone only (vs smartphone) decreased video use (−20.1 pp, 95% CI −33.5 to −6.8). Shorrocks-Shapley decomposition revealed that digital access and eHealth behaviors explained 40.4% of variance in video vs audio choice and 33.4% of video vs none; geography explained 40.5% of audio vs none; digital factors (25.7%), geography (19.7%), and health status and needs (15.5%) all contributed substantially to any vs none.

**Conclusions:**

Digital access and eHealth behaviors collectively explain more variance in modality choice than traditional sociodemographic factors. Telehealth uptake reflects a combination of digital factors, geography, and clinical need, whereas video modality specifically hinges on digital readiness. Interventions pairing sustained insurance coverage with targeted investments in device access, affordable high-speed connectivity, and digital literacy training are most likely to narrow persistent telehealth gaps.

## Introduction

The landscape of health care delivery in the United States has been reshaped by the expansion of telehealth—the use of electronic information and telecommunications technologies to support clinical care, education, and administration [1-3]. Services delivered by telephone, video, remote monitoring, or asynchronous platforms can lower geographic and logistical barriers to care [3]. However, as telehealth has scaled, concerns persist that the “digital divide” may reproduce or even widen disparities in access and outcomes [4-9].

Evidence documents persistent differences in telehealth access across sociodemographic groups. Older adults, racial and ethnic minorities, individuals with lower educational attainment, and those residing in rural areas consistently demonstrate lower telehealth use rates [10]. A systematic review found that telehealth adoption during the pandemic was significantly lower among Black and Hispanic patients compared to White patients after accounting for clinical and geographic factors [11]. Recent analyses of post-pandemic telehealth patterns reveal that while overall use has stabilized, disparities by age, income, and digital literacy persist [12]. Critically, modality choice—whether patients use video or audio-only visits—varies substantially by socioeconomic status (SES) and digital access, with audio-only visits more common among older, lower-income, and rural populations [13].

Use of telehealth surged during the initial phase of the COVID-19 public health emergency (PHE) and then declined from its peak, but it has stabilized at levels substantially higher than before the pandemic. For example, among Medicare fee-for-service beneficiaries, telehealth use in 2021 was approximately 38-fold higher than prepandemic levels (from 0.1% in 2019 to 3.8% of visits in 2021) [14]. By late 2022, about 15% of Medicare beneficiaries received telehealth in a given quarter, and in the last quarter of 2023, more than 1 in 10 beneficiaries (ie, 13%) used telehealth at least once, well above the pre-2020 baseline [15,16]. Also, in population-based surveys, 37% of US adults reported telemedicine use in 2021 and 43% of adults with a health care visit reported telemedicine use in 2022 (70% video; 30% audio) [17,18]. Taken together, these data indicate that telehealth has become a substantial component of routine care, with meaningful variation by modality (video vs audio).

The PHE also saw major federal investments in household internet affordability that intersect directly with telehealth access. The Affordable Connectivity Program (ACP) launched at the end of 2021 expanded subsidized broadband, which increased adoption among lower-income households and provided a discount for eligible households and was active throughout 2022 [19,20]. By the end of 2022, roughly 15.4 million households had enrolled (around 28% of eligible households), growing to more than 23 million by the February 2024 enrollment freeze [21,22]. The ACP ended in June 2024 when Congress did not appropriate additional funding, potentially affecting an estimated 23 million households [23]. Evidence after the ACP wind-down suggests that broadband affordability shocks can translate into foregone telehealth among subsidy-eligible households: in a post-ACP consumer survey of over 68,000 lifeline and/or former ACP participants fielded in November 2024, 36.1% of respondents who were previously enrolled in ACP reported being unable to continue with telehealth, rehabilitation, or remote medical monitoring without ACP [24]. These policy changes have altered the digital access landscape in ways that may affect who can use—and how they use—telehealth.

The digital divide is multidimensional, encompassing access to devices and connectivity, the quality and affordability of home internet, and the skills and engagement required to use digital tools effectively [25]. These dimensions map directly onto telehealth modality: video visits typically require higher-bandwidth connectivity, compatible devices, and greater comfort with app-based workflows, whereas audio-only visits may remain feasible when bandwidth, device capability, or digital skills are constrained [26,27]. However, much of the existing literature emphasizes individual predictors without quantifying the relative explanatory importance of digital, geographic, clinical, and socioeconomic domains. A domain-level approach can help distinguish which levers are most policy-relevant for improving overall uptake vs enabling equal access to video-based care.

Despite growing research on telehealth disparities, significant gaps remain in understanding the relative importance of different determinant domains. Most existing studies examine individual predictors in isolation rather than quantifying how much each domain—demographics, SES, health needs, digital access, and geography—contributes to explaining telehealth use patterns. Furthermore, few studies have simultaneously examined predictors of both any telehealth use and modality choice (video vs audio-only) in the post-PHE period, when several temporary telehealth flexibilities required repeated legislative extensions and telehealth patterns stabilized into new norms.

We use nationally representative data from the sixth cycle of the Health Information National Trends Survey (HINTS 6), administered in 2022 by the National Cancer Institute, to describe contemporary patterns of telehealth use (phone or video) and to evaluate how sociodemographic, clinical, area-level and geographic measures, psychosocial, and digital access factors relate to (1) any telehealth use and (2) modality choice. Using the Shorrocks-Shapley decomposition models, we quantify the relative contribution of each explanatory domain to explained variance in telehealth outcomes [28].

Our objectives were to (1) estimate national prevalence of telehealth use by modality (video, audio-only, and none) among US adults in 2022; (2) identify individual and contextual determinants of telehealth use and modality choice using survey-weighted regression models; and (3) quantify the relative contribution of digital access or eHealth behaviors, geography, clinical need, and socioeconomic factors to explained variance using the Shorrocks-Shapley decomposition.

## Methods

### Research Design

This study used a cross-sectional observational design using secondary analysis of nationally representative survey data linked to area-level contextual measures. The primary aim was to identify individual and contextual determinants of telehealth use and modality choice among US adults in 2022. We report this cross-sectional observational study in accordance with the STROBE (Strengthening the Reporting of Observational Studies in Epidemiology) guidelines for cross-sectional studies [29].

### Participants

#### Inclusion and Exclusion Criteria

The study population included civilian, noninstitutionalized adults aged 18 years and older residing in the United States. Eligibility was determined by the HINTS sampling frame, which excluded individuals living in institutionalized settings (eg, prisons and nursing homes), those without a valid mailing address, and residents of US territories. For this analysis, we included all respondents with valid responses to the telehealth use question.

#### Sampling Procedures

We analyzed the HINTS 6, a nationally representative survey of civilian US adults administered by the National Cancer Institute from February 2022 through November 2022 [30,31]. HINTS uses a complex 2-stage stratified random probability sampling design: first, a stratified sample of addresses was selected from a comprehensive address file, then 1 adult per household was randomly selected to complete the survey. The survey provides person-level sampling weights and 50 jackknife replicate weights to allow reliable national inference. The restricted-use version of HINTS (available to researchers through a data use agreement with the National Cancer Institute) contains geocoding variables (county Federal Information Processing Series [FIPS] codes) not included in the public-use file, enabling linkage with area-level datasets.

#### Sample Size

The HINTS 6 cycle included 6252 respondents. The primary analytic sample for telehealth modality outcomes included respondents with nonmissing telehealth modality responses (n=6046). This sample size provides adequate statistical power to detect small-to-medium effect sizes (Cohen *d* ≥0.10) with 80% power at *α*.05 for the primary regression analyses, accounting for the design effect of the complex survey design.

#### Data Linkage

Consistent with prior literature, we appended area-level measures to each respondent via county FIPS codes. County socioeconomic context for 2022 (total population, poverty rate, unemployment rate, percent without a high school diploma, and percent aged ≥65 y) was derived from the American Community Survey [32]. County-level broadband availability was obtained from the National Institute on Minority Health and Health Disparities (NIMHD) health disparities (HD) Pulse portal as the percent of residents with broadband access [33]. We additionally obtained the Centers for Disease Control and Prevention/Agency for Toxic Substances and Disease Registry (CDC/ATSDR) Social Vulnerability Index (SVI) to characterize local vulnerability [34].

### Ethical Considerations

This study analyzed the 2022 HINTS 6 restricted-use deidentified dataset. The University of Arkansas for Medical Sciences Institutional Review Board determined this study did not constitute human participants research (protocol 297801). The original HINTS data collection was approved by the Westat Institutional Review Board, and all survey respondents provided implied informed consent by completing and returning the questionnaire. The restricted-use dataset contained no direct identifiers; geographic information was limited to county-level codes. Respondents received no compensation for the secondary analysis; compensation for the original HINTS survey included a US $2 preincentive and an opportunity for a US $25 postsurvey incentive. No images containing identifiable individuals were included in this study.

### Measures

#### Outcome Variable

Telehealth exposure in the 12 months preceding the survey was categorized into 3 mutually exclusive groups—video, audio-only, and no telehealth—yielding 1641 (27.2%, 95% CI 25.5%‐29.1%), 876 (12.1%, 95% CI 10.9%‐13.4%), and 3529 (60.7%, 95% CI 58.6%‐62.7%) respondents, respectively (Table 1). Respondents reporting any video visit were assigned to the video group (even if they also had phone visits); audio-only indicates telephone visits with no video exclusively. We used “telehealth” to denote synchronous video or phone visits. Other digital health activities (apps, portals, and information seeking) were considered model predictors, not modalities of telehealth delivery.

**Table 1.** Descriptive characteristics by telehealth modality (video, audio only, and none) among US adults^a^.

Characteristics	Video	Audio only	No telehealth
Participants, n (%)	1641 (27.2)	876 (12.1)	3529 (60.7)
Age (y), mean (SD)	52.84 (16.46)	58.57 (17.55)	56.26 (17.58)
Female, n (%)	1014 (65.3)	532 (64.6)	1919 (57.0)
High school graduation, n (%)	1499 (96.7)	762 (92.1)	3120 (92.5)
Race and ethnicity, n (%)			
Asian	51 (3.1)	29 (3.3)	116 (3.3)
Black	241 (14.7)	135 (15.4)	551 (15.6)
Hispanic	262 (16)	177 (20.2)	542 (15.4)
White	1155 (76.5)	566 (71.7)	2353 (72.6)
Other	169 (10.3)	119 (13.6)	422 (12)
Metropolitan status, n (%)			
Large central metro	685 (41.7)	355 (40.5)	1221 (34.6)
Large fringe metro (suburban)	393 (23.9)	176 (20.1)	769 (21.8)
Medium or small metro	401 (24.4)	250 (28.5)	1016 (28.8)
Micropolitan	96 (5.9)	55 (6.3)	307 (8.7)
Noncore (rural)	66 (4)	40 (4.6)	216 (6.1)
Income group (US $, n (%)			
Low ($20,000)	213 (14.4)	155 (20)	555 (17.5)
Middle (20,000-75,000)	557 (37.6)	351 (45.2)	1457 (45.9)
High (≥75,000)	712 (48.0)	270 (34.8)	1161 (36.6)
Employed, n (%)	866 (55.6)	349 (41.9)	1725 (51.2)
Cancer history, n (%)	258 (15.7)	134 (15.3)	487 (13.8)
Hypertension, n (%)	731 (44.5)	452 (51.6)	1477 (41.9)
Disabled, n (%)	172 (11)	96 (11.5)	211 (6.3)
PROMIS^b^ Isolation T-score, mean (SD)	47.11 (9.97)	46.36 (9.46)	45.40 (9.30)
PROMIS Meaning T-score, mean (SD)	54.16 (10.29)	55.10 (9.75)	55.61 (10.08)
Health Insurance, n (%)	1560 (95.1)	813 (92.8)	3167 (89.7)
Health numeracy, n (%)			
Easy or very easy	1241 (75.6)	624 (71.2)	2495 (70.7)
Hard or very hard	369 (22.5)	231 (26.4)	951 (26.9)
Use of health or wellness apps, n (%)			
Used app	1103 (67.2)	414 (47.3)	1441 (40.8)
Did not use or no apps or no smart device	498 (30.3)	385 (43.9)	1871 (53)
Sought health information online, n (%)			
No	128 (7.8)	95 (10.8)	506 (14.3)
Yes	1367 (83.3)	586 (66.9)	2295 (65.0)
Not internet user	120 (7.3)	153 (17.5)	586 (16.6)
Satisfaction with home internet for health needs, n (%)			
Satisfied (extremely or very)	1074 (65.4)	439 (50.1)	1790 (50.7)
Somewhat	354 (21.6)	194 (22.1)	799 (22.6)
Not satisfied or nonuser	185 (11.3)	207 (23.6)	802 (22.7)
Device ownership, n (%)			
Any smart device (tablet or smartphone or multiple)	1582 (96.4)	750 (85.6)	3092 (87.6)
No smart device (basic cell only or none)	54 (3.3)	120 (13.7)	417 (11.8)
Census division, n (%)			
New England	80 (4.9)	39 (4.5)	108 (3.1)
Middle Atlantic	192 (11.7)	83 (9.5)	399 (11.3)
East North Central	170 (10.4)	98 (11.2)	481 (13.6)
West North Central	51 (3.1)	17 (1.9)	210 (6)
South Atlantic	444 (27.1)	204 (23.3)	878 (24.9)
East South Central	51 (3.1)	39 (4.5)	202 (5.7)
West South Central	232 (14.1)	117 (13.4)	567 (16.1)
Mountain	126 (7.7)	58 (6.6)	255 (7.2)
Pacific	295 (18)	221 (25.2)	429 (12.2)

a A fully corrected Table S1 is provided as a supplementary file (Multimedia Appendix 1) in PDF and LaTeX source formats, with the corrected values shown in bold and all percentages reported to one decimal place.

b PROMIS: Patient-Reported Outcomes Measurement Information System.

#### Explanatory Variables

A comprehensive set of explanatory variables to explain telehealth use was constructed based on prior literature on the digital divide and health care access [7,30,31,35]. Variable definitions and coding for individual-level demographics, SES, health status, digital access and literacy (device ownership, satisfaction with home internet for health needs, health numeracy, health or wellness app use, and online health information seeking), and geographic indicators (US Census division and urbanicity) are summarized in Appendix A in Multimedia Appendix 1.

#### Digital Readiness Variables

We modeled digital readiness using separate indicators for device ownership, internet satisfaction, health app use, and online health information seeking rather than combining them into a single composite score via factor analysis. This approach was chosen for 3 reasons. First, these constructs are theoretically distinct: device ownership reflects material access, internet satisfaction captures infrastructure quality, and app use or information seeking represents behavioral engagement and digital skills [36]. Second, separate coefficients provide policy information—interventions targeting device distribution differ from those promoting digital literacy or improving broadband infrastructure [37]. Finally, the Shorrocks-Shapley decomposition explicitly partitions explained variance across covariate domains, allowing us to quantify the relative importance of “digital access” vs “health literacy and eHealth” without collapsing distinct mechanisms into a single latent factor.

#### Psychosocial Variables

HINTS 6 includes validated Patient-Reported Outcomes Measurement Information System (PROMIS) short forms measuring psychosocial constructs [38,39]. The PROMIS Social Isolation 4-item short form assesses perceptions of being excluded, disconnected, or isolated from others; scores are reported as T-scores (population: mean 50, SD 10), with higher scores indicating greater isolation. The PROMIS Meaning and Purpose 4-item short form assesses sense of meaning, purpose, and engagement in life, with higher T-scores indicating greater sense of purpose.

PROMIS measures were included as exploratory covariates to assess whether psychosocial context contributed meaningfully beyond digital, geographic, clinical, and socioeconomic domains. Social isolation has been associated with reduced health care use, lower technology adoption, and barriers to accessing care [40,41]. Conversely, individuals with a greater sense of meaning and purpose may be more motivated to engage with health services and adopt health-promoting technologies [42]. These constructs may therefore influence both the decision to use telehealth and the choice of modality, independent of traditional sociodemographic and health-related factors.

### Analytic Strategy

#### Primary Analyses

We report descriptive statistics and estimate multivariable associations using survey-weighted linear probability models (LPMs) for four contrasts: (1) video vs audio-only, (2) video vs no telehealth, (3) audio-only vs no telehealth, and (4) any telehealth (video or audio) vs no telehealth. The LPM directly models the probability of the outcome as a linear function of covariates and with survey weights provides easily interpretable absolute risk differences in percentage points [43,44]. Coefficients are interpreted as the percentage-point (pp) change in the probability of the outcome for a 1-unit change in the predictor, holding other variables constant.

#### Survey Design Adjustments

All adjusted analyses incorporated the complex survey design of HINTS 6. We used the final person-level weight and the 50 jackknife replicate weights to obtain design-consistent point estimates and robust standard errors, following HINTS technical guidance [45]. This approach yields nationally representative estimates with SEs and *P* values adjusted for stratification and clustering. Replicate-weight variance estimation used the jackknife mean squared error (MSE).

#### Decomposition Analysis

To quantify the explanatory contribution of prespecified covariate domains, we applied a Shorrocks-Shapley decomposition of the model *R*^2^, partitioning the total *R*^2^ into additive contributions from groups of predictors (demographics, SES, health status and needs, health system access, geography, urbanicity, psychosocial factors, digital access, health literacy and eHealth, and area context) [28,46]. Decompositions were implemented for each survey-weighted LPM using the *shapley2* module in Stata (StataCorp LLC) [47]. The Shorrocks-Shapley approach does not imply causal ordering or construct hierarchy; it quantifies predictive importance.

#### Missing Data Analysis

We evaluated item-level missingness across all study variables prior to model estimation. Missingness was assessed for continuous measures (including PROMIS T-scores and self-rated health) and for sociodemographic covariates (including employment status, disability status, race or ethnicity, and household income). To minimize loss of observations in adjusted models and to preserve the survey sample structure, selected categorical covariates with modest item nonresponse and/or structurally nonapplicable responses (indicators related to digital access and online behaviors) were coded with an explicit “missing/unknown” category. These missingness categories were included as indicator levels in the regression models to avoid listwise deletion for those variables, and coefficients for missingness indicators were not interpreted substantively.

To evaluate the plausibility of common missing-data mechanisms, we first applied the Little Missing Completely at Random (MCAR) test [48]. In this way, we assessed whether observed values differed systematically across missing data. Second, because income exhibited the largest item nonresponse among key sociodemographic measures, we performed a supplementary missingness model in which an indicator for income nonresponse was regressed with minimal missingness (age, census division, cancer history, and insurance status). These diagnostics were used to guide the selection of an appropriate missing-data handling strategy for sensitivity analyses.

As a sensitivity analysis, we conducted multiple imputation by chained equations under a missing at random (MAR) framework conditional on observed data. We generated *m*=20 imputed datasets. Variables imputed included household income, race or ethnicity, employment status, disability status, self-rated health, sex, marital status, education, and PROMIS psychosocial measures. The imputation models incorporated all variables used in the primary analyses, along with auxiliary predictors expected to be associated with missingness and/or the imputed values (including age, census division, urbanicity, cancer history, high blood pressure, and insurance status). After imputation, we re-estimated the primary survey-weighted regression models in each imputed dataset and pooled point estimates and SEs across imputations using standard combining rules. We then compared pooled estimates from the imputed analyses with the complete-case results to evaluate robustness; full imputation model specifications, diagnostics, and pooled regression outputs are provided in Multimedia Appendix 1.

#### Software: Statistical Threshold

All analyses were conducted in Stata/MP 18 (StataCorp LLC) using survey procedures (svy: prefix) with the provided jackknife replicate weights and MSE option, consistent with HINTS methodological guidelines. Tests were 2-sided with an *α* of .05.

## Results

### Sample Characteristics by Telehealth Modality

Table 1 summarizes characteristics by telehealth modality. Among 6252 respondents in HINTS 6, 1641 reported any video telehealth, 876 reported exclusively audio-only telehealth, and 3529 reported no telehealth in the past 12 months. These correspond to survey-weighted prevalence of 27.2% (95% CI 25.5%‐29.1%), 12.1% (95% CI 10.9%‐13.4%), and 60.7% (95% CI 58.6%‐62.7%), respectively. Video users were younger on average (52.8, SD 16.5 y) than those with no telehealth (56.3, SD 17.6 y) and audio-only users (58.6, SD 17.6 y). Women comprised a larger share of video (1014/1554, 65.3%) and audio-only (532/824, 64.6%) users than nonusers (1919/3365, 57%). Compared with nonusers, video users more frequently reported higher income (712/1482, 48% vs 1161/3173, 36.6% at ≥US $75,000), current employment (866/1557, 55.6% vs 1725/3371, 51.2%), and insurance coverage (1560/1641, 95.1% vs 3167/3529, 89.7%). Digital readiness was notably higher among video users: any smart device ownership (1582/1641, 96.4% vs 3092/3529 87.6%), health or wellness app use (1103/1641, 67.2% vs 1441/3529, 40.8%), and online health information seeking (1367/1641, 83.3% vs 2295/3529, 65%). Satisfaction with home internet for health needs was also higher in the video group (1074/1641, 65.4% extremely or very satisfied vs 1790/3529, 50.7% among nonusers). Geographic composition differed across modalities, with video visits more common among residents of large central metros (685/1641, 41.7%) and large fringe metro or suburban areas (393/1641, 23.9%) compared with nonusers (1221/3529, 34.6% and 769/3529, 21.8%, respectively). White respondents made up a similar share across video, audio-only, and no telehealth groups, while Hispanic respondents were somewhat more represented among audio-only users than among video users or those who did not use telehealth.

### Regression Estimates

#### Key Predictors

Table 2 reports key predictors from survey-weighted LPMs for all 4 contrasts, presenting marginal effects as pp differences. Below we summarize the key patterns; full coefficient estimates and CIs for all covariates appear in Tables S5-S8 in Multimedia Appendix 1.

**Table 2.** Key predictors from survey-weighted linear probability models of telehealth outcomes, Health Information National Trends Survey (HINTS 6)^a^.

Variables	Video vs audio^b^ (n=2069)	Video vs none^c^ (n=4317)	Audio vs none^d^ (n=3632)	Any vs none^e^ (n=5009)
Demographics and socioeconomics				
Male	−0.2 (−5.5 to 5.2; *P*=.94)	−8.5 (−12.4 to −4.6; *P<*.001)	−5.5 (−8.1 to −2.8; *P<*.001)	−9.7 (−14.0 to −5.4; *P<*.001)
White	2.4 (−3.4 to 8.2; *P*=.42)	6.4 (2.3 to 10.6; *P=0.001*)	3.7 (0.7 to 6.7; *P*=.02)	7.1 (2.2 to 12.0; *P<0.001*)
Employed	9.1 (2.6 to 15.5; *P=0.001*	3.8 (−1.6 to 9.2; *P*=.17)	−3.3 (−6.8 to 0.2; *P*=.07)	0.9 (−3.3 to 5.1; *P*=.67)
Health status and access				
Disabled	9.6 (1.6 to 17.6; *P*=.02)	23.7 (17.8 to 29.6; *P<*.001)	11.9 (5.1 to 18.7; *P<*.001)	22.5 (16.1 to 28.8; *P<*.001)
High blood pressure	−1.7 (−7.8 to 4.3; *P*=.58)	5.8 (1.8 to 9.8; *P=0.001*)	5.8 (2.6 to 8.9; *P<*.001)	8.0 (4.8 to 11.2; *P<*.001)
Health insurance	21.2 (13.0 to 29.3; *P<*.001)	13.3 (9.2 to 17.3; *P<*.001)	1.1 (−4.0 to 6.3; *P*=.67)	11.5 (4.6 to 18.4; *P*=.001)
Digital access				
Basic cell only (reference: landline or none)	−20.1 (−33.5 to −6.8; *P=0.001*)	−7.4 (−16.4 to 1.7; *P*=.11)	2.6 (−3.9 to 9.1; *P*=.44)	−4.0 (−10.6 to 2.6; *P*=.23)
Internet: extremely satisfied	8.6 (1.3 to 15.9; *P*=.02)	7.7 (1.1 to 14.4; *P*=.02)	0.1 (−5.4 to 5.6; *P*=.97)	5.1 (−1.3 to 11.5; *P*=.12)
eHealth behaviors				
Used health apps	5.5 (−3.4 to 14.5; *P*=.23)	17.5 (12.5 to 22.6; *P<*.001)	9.1 (5.5 to 12.7; *P<*.001)	18.4 (12.0 to 24.8; *P<*.001)
Seeks health information online	6.6 (−5.5 to 18.7; *P*=.28)	7.2 (1.0 to 13.4; *P*=.02)	1.7 (−1.0 to 4.4; *P*=.22)	7.2 (2.4 to 12.0; *P=0.001*)

a Coefficients represent percentage-point differences in probability of outcome (95% CI; *P* value). Models additionally controlled for: age, marital status, income, education, self-rated health, cancer history, census division, urbanicity, Patient-Reported Outcomes Measurement Information System (PROMIS) isolation and meaning scores, other device categories, internet satisfaction levels, health numeracy, and county-level characteristics.

b *R*2 for “video vs audio” is 0.08.

c *R*2 for “video vs none” is 0.13.

d *R*2 for “audio vs none” is 0.07.

e *R*2 for “any vs none” is 0.12.

#### Demographics and Socioeconomic Factors

Gender differences were consistent: men were 8 to 10 pp less likely than women to use telehealth across all uptake contrasts (*P*<.001 for video and any telehealth vs none). White respondents had 4 to 7 pp higher use than non-White respondents, but race or ethnicity was not significantly associated with video vs audio modality choice. Adults aged 35 to 49 years showed elevated telehealth use (approximately 6‐7 pp) compared with older age groups. Among telehealth users, employment and insurance coverage were strongly associated with choosing video over audio (9.1 pp and 21.2 pp, respectively).

#### Health Status and Needs

Health-related factors showed the largest effect sizes. Disability was strongly associated with telehealth use: 22 to 24 pp for video and any telehealth vs none, and 12 pp for audio-only vs none. Among telehealth users, disability was also associated with choosing video over audio (9.6 pp). Cancer history (6‐7 pp) and hypertension (6‐8 pp) were positively associated with all forms of telehealth uptake, but were not significant predictors of choosing video vs audio. Insurance coverage was consistently positive (11‐13 pp for uptake; 21 pp for video vs audio choice).

#### Digital readiness

Digital factors differentiated video from audio use. Basic cell phone only (vs smartphone or tablet) was associated with 20 pp lower video use among telehealth users. Internet satisfaction strongly favored video: “extremely” or “very satisfied” users were 8 to 9 pp more likely to choose video over audio. Health app use showed the largest digital association with uptake: 17 to 18 pp for video and any telehealth vs none. Online health information seeking was positively associated with video (7 pp) but not audio-only uptake.

#### Geographic Variation

Geography was especially salient for audio-only use. Relative to New England, audio-only use was 9 to 19 pp lower across multiple census divisions (East North Central, West North Central, South Atlantic, and West South Central). For any telehealth vs none, the West North Central showed the largest deficit (−21 pp). Geographic variation for video use was more modest, with only West North Central showing a significant deficit (−13 pp vs New England).

#### Psychosocial Factors

PROMIS measures showed small associations. Social isolation was weakly associated with any telehealth use (0.2 pp per T-score point). Among telehealth users, greater meaning in life was associated with lower video use (−0.3 pp per point), though this effect was small in magnitude.

### Shorrocks-Shapley Decomposition

#### Explained Variation and Shapley Decomposition

Model fit, expressed as Shapley-based *R*^2^, was modest and varied by contrast: 0.08 (video vs audio), 0.13 (video vs none), 0.07 (audio vs none), and 0.12 (any vs none; Table 3). Decomposition of *R*^2^ across prespecified domains highlighted distinct drivers for modality choice vs telehealth uptake. For video vs audio, digital access was the leading contributor (25.1% of explained variance), followed by SES (23.5%), health literacy and eHealth (15.3%), and geography (13.8%). Together, digital access+health literacy and eHealth accounted for 40.4% of explained variance, underscoring that device ownership, internet satisfaction, and online engagement are central to video over audio.

**Table 3.** Shapley decomposition of survey-weighted linear probability models (LPMs)^a^.

Variable group	Video vs audio-only, *R*^2^ (%)		Video vs no telehealth, *R*^2^ (%)		Audio-only vs no telehealth, *R*^2^ (%)		Any telehealth vs no telehealth, *R*^2^ (%)	
Demographics	0.0029 (3.65)		0.0164 (12.61)		0.0036 (5.13)		0.0142 (11.87)	
Socioeconomic status	0.0188 (23.49)		0.0154 (11.83)		0.0049 (7.03)		0.0095 (7.91)	
Health status and needs	0.0057 (7.12)		0.0153 (11.79)		0.0148 (21.08)		0.0186 (15.53)	
Health system access	0.0030 (3.80)		0.0065 (5.00)		0.0013 (1.83)		0.0057 (4.76)	
Geography	0.0110 (13.78)		0.0158 (12.15)		0.0283 (40.49)		0.0237 (19.71)	
Urbanicity	0.0011 (1.34)		0.0042 (3.25)		0.0013 (1.91)		0.0038 (3.19)	
Psychosocial factors	0.0016 (1.95)		0.0104 (7.97)		0.0011 (1.53)		0.0088 (7.34)	
Digital access	0.0201 (25.07)		0.0186 (14.31)		0.0010 (1.36)		0.0099 (8.23)	
Health literacy and eHealth	0.0122 (15.27)		0.0248 (19.06)		0.0062 (8.79)		0.0209 (17.45)	
Area context	0.0036 (4.52)		0.0026 (2.03)		0.0076 (10.85)		0.0048 (4.03)	
Total	0.08 (100)		0.13 (100)		0.07 (100)		0.12 (100)	

a Shapley decomposition apportions the total *R*2 of each model into the marginal contributions from distinct groups of predictor variables. All models were survey-weighted. Variable groups defined as (1) demographics: gender, marital status, age group, and race or ethnicity (White vs non-White); (2) socioeconomic status: household income, education level, and employment status; (3) health status and needs: disability status, self-rated health, cancer history, and high blood pressure history; (4) health system access: insurance status; (5) geography: US Census division; (6) urbanicity: urban, suburban, and rural; (7) psychosocial factors: Patient-Reported Outcomes Measurement Information System (PROMIS)—”Social Isolation” and “Meaning and Purpose” scores; (8) digital access: device ownership, satisfaction with home internet for health needs, and county-level percent broadband access; (9) health literacy and eHealth: self-reported health numeracy, use of health or wellness apps, and online health information-seeking behavior; and (10) area context: county-level characteristics including total population, poverty rate, unemployment rate, percent without a high school diploma, and percent of population aged ≥65 years.

For video vs no telehealth, the pattern remained digitally oriented but more diffuse: health literacy and eHealth (19.1%) and digital access (14.3%) ranked highly, as did demographics (12.6%), geography (12.2%), SES (11.8%), and health status and needs (11.8%). The digital stack contributed 33.4%, while demographics and SES jointly explained 24.4%, and geography, urbanicity, and area context explained 17.4%.

For audio-only vs no telehealth, the leading domain shifted decisively to place: geography alone explained 40.5%, followed by health status and needs (21.1%) and area context (10.9%). The digital stack was comparatively small (10.2%), while the aggregate place–based share reached 53.2%, indicating that where people live (division and local socioeconomic context) is especially salient for audio uptake. Finally, for any telehealth vs none, contributions were balanced across domains: geography (19.7%) and health literacy and eHealth (17.5%) led, followed by health status and needs (15.5%) and demographics (11.9%). The digital stack accounted for 25.7%, place-based factors for 26.9%, and demographics+SES for 19.8%. Across results, insurance and urbanicity contributed modest shares, whereas psychosocial factors contributed minimally (2%) for video vs audio but were more substantial for video vs none (8%) and any vs none (7.3%), suggesting a limited role in modality choice but some role in overall uptake.

#### Missing Data Patterns and Multiple Imputation Sensitivity Analysis

Item-level missingness varied across variables. Key continuous variables had moderate missingness: PROMIS Isolation T-scores (n=334, 5.3%), PROMIS Meaning T-scores (n=314, 5%), and self-rated health (n=234, 3.7%). Sociodemographic variables with missingness included: employment status (n=390, 6.2%), disability status (n=390, 6.2%), race or ethnicity (n=619, 9.9%), and income (n=732, 11.7%). For categorical variables with modest missingness, we used a “missing-as-its-own-category” approach: cancer history (5.9% missing or unknown), digital access indicators (0.6%‐14.8% missing or not applicable), and health app use or online health information seeking (3.7%‐5.8% missing).

The null hypothesis of the MCAR test [48] was rejected (*χ*^2^_530_=982.1; *P*<.001), indicating that data were not MCAR. As a supplementary analysis, we conducted logistic regression examining whether missingness on income (the variable with the highest missingness) was predicted by fully observed covariates; this analysis also indicated systematic missingness (*χ*^2^_1_=1354.6, *P*<.001), with missingness associated with age, census division, cancer history, and insurance status. Together, these results indicate that missingness is related to observed covariates. Accordingly, we conducted multiple imputation by chained equations under a MAR assumption as a recommended sensitivity approach for item nonresponse in observational studies [49,50].

#### Multiple Imputation Sensitivity Analysis

Given the MAR finding, we conducted a sensitivity analysis using multiple imputation by chained equations with *m*=20 imputations. Imputed variables included income, race, employment, disability, self-rated health, gender, marital status, education, and PROMIS scores. Imputation models included all analysis variables plus auxiliary predictors (age, census division, urbanicity, cancer history, high blood pressure, and insurance status). Results from multiple imputations analyses were consistent with complete-case estimates, supporting the robustness of our primary findings (see Table S1 in Multimedia Appendix 1). Complete multiple imputations regression results with 95% CIs are provided in Tables S2–S4 and accompanying data files in Multimedia Appendix 1.

## Discussion

### Principal Findings

In a nationally representative survey of US adults in 2022, we found that overall telehealth use (any vs none) showed large regression effect sizes for health needs (eg, disability, +22.5 pp) and insurance coverage (+11.5 pp), whereas video vs audio-only modality choice among telehealth users was most strongly differentiated by digital readiness (device access and satisfaction with home internet) and eHealth behaviors. Shorrocks-Shapley decomposition revealed distinct variance patterns: for any telehealth vs none, geography (19.7%), digital factors (25.7%), and health status (15.5%) all contributed substantially; digital domains explained the largest share of variation in video vs audio choice (40.4%), while geography and area context contributed most to explaining audio-only uptake (53.2% combined for audio vs none). Men were less likely than women to use telehealth, consistent with gender differences in health care utilization [51], and telehealth users with limited device capability were more likely to rely on audio-only visits. Hypertension was positively associated with telehealth uptake but not with video vs audio choice, potentially reflecting that patients with chronic conditions requiring frequent visits benefit from any telehealth modality without a strong preference for video.

This study advances prior work in 3 ways. First, we distinguish modality choice (video vs audio) from uptake (any telehealth vs none) in a unified, survey-weighted framework, showing that digital access+health literacy and eHealth domains together explain over 40% of the variance in video vs audio choice (40.4%) and one-third in video vs none (33.4%). Second, we explicitly separate individual digital behaviors (app use and health information seeking) from device ownership and satisfaction, demonstrating that behavioral engagement is strongly linked to uptake above and beyond device availability. Third, we link person-level data with area context (division, urbanicity, and county characteristics) and apportion explained variance across domains using a Shorrocks-Shapley decomposition adapted to complex surveys—offering decision makers or researchers a transparent view of the relative leverage points for potential interventions.

A key strength of this study relative to the telehealth disparities literature is the granularity of the person-level technology data available in HINTS 6, which allows us to operationalize digital readiness beyond proxies such as binary internet access or area-level broadband coverage alone. Specifically, HINTS captures multiple, policy-relevant dimensions of digital readiness—device access and ownership, perceived adequacy of home internet for meeting health-related needs, and behavioral engagement with digital health. We leveraged this richness in a two-level way to avoid inconsistent aggregation: (1) we modeled these measures as distinct indicators in the regression analyses to preserve interpretability and map directly onto different intervention levers, and (2) we grouped them into prespecified domains in the Shorrocks-Shapley decomposition to provide a coherent summary of the relative explanatory contribution of the broader digital readiness “stack” compared with sociodemographic, clinical, and place-based domains. Future work could apply psychometric methods to develop and validate a composite digital readiness scale. In contrast, our indicator plus–domain approach prioritizes actionable interpretability for policy or practice [52,53].

The decomposition analysis shows 3 patterns. First, the digital access+health literacy and eHealth consistently explain a large fraction of variation, but its weight is contrast-specific: it is largest for video vs audio and video vs none, smaller for any vs none, and smallest for audio vs none. Second, geography+urbanicity+area context is most influential for audio vs none, moderate for any vs none and video vs none, and least for video vs audio. Third, the combined health status and needs+health system access contribution is largest when the contrast is framed as use vs nonuse (audio vs none and any vs none), and smaller when the choice is video vs audio. Finally, because the effect size for social isolation was small and imprecise across modality contrasts, we interpret this as suggestive at best. It may reflect unmeasured factors (eg, mobility or transport barriers or care preferences) correlated with isolation rather than a direct effect.

Taken together, these patterns imply that (1) modality is largely a function of digital readiness and skills, while (2) adoption (any use vs none) is a joint function of clinical need, geography or place, and digital readiness. Despite modest *R*^2^—typical for behavioral use models—the decomposition points to clear, policy-addressable levers: device access and digital engagement for video-based care; and place-based strategies (alongside coverage and need) to broaden overall telehealth uptake.

A prior nationwide study in older adults shows that living in a neighborhood at the lowest tertile of broadband internet subscription is associated with a significantly lower likelihood of using telehealth [54]. We similarly observe strong associations between digital readiness and video use. Lacking a smart device is strongly associated with choosing audio over video but was not significantly associated with any telehealth vs none in adjusted models. In uptake models, app use and online health information seeking are positive, whereas device category is not significantly associated with any telehealth vs none. However, our adjusted county-level percent broadband access shows small associations with any and audio telehealth. This likely reflects differences between availability vs quality or speed and aggregation by counties, which might dilute neighborhood heterogeneity. Together, the findings underscore that closing gaps requires more than network presence; adoption and affordability are important. Our modality-specific results align: device richness and internet satisfaction push use toward video over audio, while those with fewer devices or lower satisfaction are more likely to rely on audio. This is consistent with a study that emphasized the importance of optimal broadband connectivity for improved mental health access and raised quality considerations as visual cues may provide useful clinical information and improve patient-clinician rapport/access to video-based care [55].

Furthermore, a recent study by Tilhou et al [56] reported that telehealth expansion exposed differences in the use of telehealth services that persisted even among beneficiaries with high-speed internet and that expansion of telehealth service or access to high-speed internet might not close gaps in use. Despite strong associations for digital access and eHealth behaviors, geography and social context still explain sizable variance. Thus, high-quality connectivity is likely important but not sufficient without attention to coverage, device access, and digital skills or engagement. Our domain-level results empirically support that digital inclusion is considered a “super” social determinant of health [37] as the device access and skills or engagement carry a substantial explanatory weight, while area context signals persistent place-based differences. The decomposition attributes important shares to geography and smaller but nontrivial shares to area context even after detailed individual controls, reinforcing that place effects remain operative for digital health adoption, consistent with a study conducted in Chicago which found that segregation and concentrated poverty influence why residents do not have home internet access [57].

### Policy and Practice Implications

Our findings point to complementary levers. First, sustaining insurance coverage and targeting populations with elevated needs (eg, disability and chronic conditions) may yield large absolute gains in telehealth use. Second, programs that upgrade device access and digital engagement (eg, app enablement and coaching in online health information seeking) should accompany broadband deployment; these address the “devices” and “skills or training” pillars alongside connectivity. Third, where home connectivity is constrained, offering high-bandwidth alternatives (community clinics and libraries) and maintaining audio-only options preserves access while longer-term infrastructure and affordability gaps are addressed. Finally, because geography and area context explain substantial variance, especially for audio telehealth–place-based investments (including affordability supports) are warranted. These implications are directly supported by our Shorrocks-Shapley decomposition, which quantifies that digital readiness is the dominant explanatory domain for video vs audio modality choice; whereas place-based factors play a larger role for audio-only uptake.

The overarching goal of telehealth is to democratize high-value care by overcoming geographic and logistical barriers. However, our findings indicate that this promise might remain unfulfilled. The significant differences identified in our nationally representative analysis reveal that telehealth, in its current form, may risk exacerbating the very differences it is intended to solve [58]. These results carry direct implications for policymakers, health care systems, and technology developers aiming to ensure the expansion of virtual care is available to all individuals.

In our data, “geography” primarily captures regional policy environments, market or provider supply for virtual care, as well as digital infrastructure and adoption. “Underserved” in this context refers to areas with lower broadband adoption or affordability and speeds, higher area deprivation, rural or micropolitan location, and limited provider supply for video-capable care. For audio only vs no telehealth, geography explained the largest share of the model’s variance—substantially more than in other contrasts—underscoring that a one-size-fits-all strategy is insufficient. The significant reductions in the probability of telehealth use by geography, for example, for residents of the Midwest and South compared to the Northeast point to potential structural issues. This aligns with research highlighting how state-level policies, particularly those governing reimbursement and physician licensure, create a fragmented regulatory landscape that hinders uniform adoption [59]. To address this, federal and state policies should prioritize targeted investments in underserved regions [60,61]. This includes not only expanding community infrastructure like broadband internet, a known barrier [60-64], but also incentivizing provider adoption through sustainable reimbursement models and advancing interstate licensure compacts to improve the supply of virtual care in these areas.

Overall, since affordability is a binding constraint for adoption, recent federal policy changes—including 2025 adjustments to Broadband Equity, Access, and Deployment program affordability requirements and the termination of Digital Equity Act grant programs—threaten to undermine gains in telehealth use, particularly in high-need regions where subsidized connectivity and digital inclusion supports have been critical [65,66].

Second, our analysis provides robust empirical weight to the emerging concept of digital determinants of health [63,64]. We found that the combined domains of health literacy and digital access are as important as geography in explaining telehealth use. Critically, this difference is not merely about access to a device but about the capability to use it effectively for health. We did not find consistent negative associations for low health numeracy after adjustment. Instead, app use and online information seeking were the behavioral factors most strongly linked to higher telehealth uptake. One interpretation is that these behaviors proxy digital readiness—familiarity with app-based health workflows similar to those required for scheduling, check-in, and joining video visits. Patient studies of video visits report that technical and setup challenges (eg, connecting to the visit, audio or video problems, and confusing initial setup) remain common and systematic reviews identify limited technical literacy and technology barriers as recurring obstacles to telehealth use in older adults. Accordingly, this pattern is hypothesis-generating and suggests that the usability burden of telehealth platforms could disproportionately deter patients with less prior digital health experience [5,8,9,67].

Overall, initiatives should address both digital access and digital literacy [68]. This requires a 2-pronged approach: funding for community-based digital health literacy programs and a push for user-centered design principles in the development of patient-facing technologies to reduce overall burden on users.

### Limitations

Cross-sectional data preclude causal inference and telehealth measures were self-reported and lacked encounter frequency or quality. County-level broadband access may mis-measure adoption, affordability, and speed (and operates at a coarser geography); collinearity with urbanicity and digital behaviors may attenuate or reverse associations for the county broadband variable. Model *R*^2^ values are modest, indicating unmeasured determinants (eg, provider supply). Future research should examine finer gradations of educational measure to distinguish groups that may face distinct barriers to telehealth adoption. Nevertheless, the survey-weighted national scope, explicit modality contrasts, and Shorrocks-Shapley decomposition across domains provide a comprehensive, policy-relevant portrait of telehealth determinants in 2022. Furthermore, understanding intersectional perspectives across race, income, education, and broadband availability might be an area of fruitful research.

### Conclusions

Video telehealth uptake in the United States reflects intersecting determinants with modality choice driven most strongly by digital readiness and overall uptake shaped by digital factors, place-based characteristics, and health needs. Policies that simultaneously advance connectivity, affordability, device access, and digital skills support are likely to narrow persistent gaps in telehealth use across modalities. Methodologically, this study advances the telehealth disparities literature by applying Shorrocks-Shapley decomposition to survey-weighted national data to quantify the relative explanatory contribution of determinant domains rather than examining predictors in isolation. Our approach provides a domain-level ranking of where disparities are most strongly concentrated, distinguishing digital readiness mechanisms from place-based constraints. Leveraging HINTS’ rich person-level technology measures, we show that digital access and eHealth behaviors account for a larger share of explained variation in video-vs-audio modality choice than traditional sociodemographic characteristics. These findings support a dual strategy: maintain reimbursable audio-only care to preserve access while digital constraints persist and pair sustained coverage with targeted investments in devices, affordable high-speed connectivity, and digital skills supports to enable more equitable access to video-based telehealth.

## Supplementary material

10.2196/81879 Multimedia Appendix 1 Supplementary materials including definition of variables, complete linear probability model results, multiple imputation sensitivity analysis, and supplementary data files.

## Abbreviations

ACP: Affordable Connectivity Program
FIPS: Federal Information Processing Series
HINTS: Health Information National Trends Survey
HINTS: Health Information National Trends Survey
LPM: linear probability model
MAR: missing at random
MCAR: missing completely at random
PHE: public health emergency
pp: percentage point
PROMIS: Patient-Reported Outcomes Measurement Information System
SES: socioeconomic status
STROBE: Strengthening the Reporting of Observational Studies in Epidemiology
