# Correction: Telehealth Use and Modality Choice Among US Adults: Shorrocks-Shapley Decomposition of a 2022 Cross-Sectional National Survey

**DOI:** 10.2196/110409

**Published:** 2026-10-15

**Authors:** Corneliu Bolbocean, Corey Hayes, Cari Bogulski

**Affiliations:** 1 Nuffield Department of Primary Care Health Sciences, University of Oxford, Radcliffe Primary Care Building, Radcliffe Observatory Quarter, Woodstock Rd, Oxford, OX2 6GG, United Kingdom, 44 1865 617855; 2 Department of Pharmacy Practice, University of Arkansas for Medical Sciences, Little Rock, AR, United States; 3 Department of Biomedical Informatics, College of Medicine, UAMS, Little Rock, AR, United States

In “Telehealth Use and Modality Choice Among US Adults: Shorrocks-Shapley Decomposition of a 2022 Cross-Sectional National Survey” [1], the authors identified errors in the descriptive statistics reported in Table 1 and the Results section.

## Corrections to Table 1

During the standardization of dual-numerator constructions, derived numerators (observed percentages rescaled to full group denominators) were retained in place of the observed unweighted counts in several instances (as shown in Table 1 of this correction notice). In addition, the Table 1 footnote misdescribed the percentages as survey-weighted.

**Table 1.** The following 16 values in 15 cells have been corrected (all other values are unchanged).

Row	Column	Originally published	Corrected
Female, n (%)	Video	1071 (65.3)	1014 (65.3)
Female, n (%)	Audio only	566 (64.6)	532 (64.6)
Female, n (%)	No telehealth	2012 (57)	1919 (57.0)
Metropolitan status: Large central metro	Video	684 (41.7)	685 (41.7)
Metropolitan status: Large central metro	No telehealth	1220 (34.6)	1221 (34.6)
Income group (US $): High (≥75,000)	Video	788 (48)	712 (48.0)
Income group (US $): High (≥75,000)	No telehealth	1292 (36.6)	1161 (36.6)
Employed, n (%)	Video	912 (55.6)	866 (55.6)
Employed, n (%)	No telehealth	1807 (51.2)	1725 (51.2)
Health insurance, n (%)	Video	1559 (95.1)	1560 (95.1)
Health insurance, n (%)	No telehealth	3165 (89.7)	3167 (89.7)
Sought health information online: Yes	No telehealth	2294 (65)	2295 (65.0)
Satisfaction with home internet: Satisfied (extremely or very)	Video	1075 (65.5)	1074 (65.4)
Satisfaction with home internet: Satisfied (extremely or very)	No telehealth	1789 (50.7)	1790 (50.7)
Device ownership: Any smart device	No telehealth	3091 (87.6)	3092 (87.6)

These corrections affect the descriptive presentation only. The analytic sample, group definitions, survey-weighted prevalence estimates (27.2%, 12.1%, and 60.7%), the Shorrocks-Shapley decomposition, all regression models (Tables 2 and 3), and all findings and conclusions are unchanged. The Results section is otherwise unchanged; only the numerals listed below have been corrected within otherwise identical sentences.

In Table 1, the following footnote has been revised:

n is unweighted and percentage is survey-weighted.

The footnote now reads:

A fully corrected Table S1 is provided as a supplementary file (Multimedia Appendix 1) in PDF and LaTeX source formats, with the corrected values shown in bold and all percentages reported to one decimal place.

## Corrections to the Results Section

Under Results, subsection Sample Characteristics by Telehealth Modality, the following sentences were corrected.

First, the following sentence was revised:

Video users were younger on average (52.8, SD 16.2 y) than those with no telehealth (56.3, SD 17.1 y) and audio-only users (58.6, SD 16.8 y).

The sentence now reads:

Video users were younger on average (52.8, SD 16.5 y) than those with no telehealth (56.3, SD 17.6 y) and audio-only users (58.6, SD 17.6 y).

The originally published SDs did not match Table 1, which correctly reports 16.46, 17.58, and 17.55.

Second, the following sentence was revised:

Women comprised a larger share of video (1071/1641, 65.3%) and audio-only (566/876, 64.6%) users than nonusers (2012/3529, 57%).

The sentence now reads:

Women comprised a larger share of video (1014/1554, 65.3%) and audio-only (532/824, 64.6%) users than nonusers (1919/3365, 57%).

Third, the following sentence was revised:

Compared with nonusers, video users more frequently reported higher income (788/1641, 48% vs 1292/3529, 36.6% at ≥US $75,000), current employment (912/1641, 55.6% vs 1807/3529, 51.2%), and insurance coverage (1559/1641, 95.1% vs 3165/3529, 90%).

The sentence now reads:

Compared with nonusers, video users more frequently reported higher income (712/1482, 48% vs 1161/3173, 36.6% at ≥US $75,000), current employment (866/1557, 55.6% vs 1725/3371, 51.2%), and insurance coverage (1560/1641, 95.1% vs 3167/3529, 89.7%).

Fourth, the following sentence was revised:

Digital readiness was notably higher among video users: any smart device ownership (1582/1641, 96.4% vs 3091/3529, 87.6%), health or wellness app use (1103/1641, 67.2% vs 1441/3529, 40.8%), and online health information seeking (1367/1641, 83.3% vs 2294/3529, 65%).

The sentence now reads:

Digital readiness was notably higher among video users: any smart device ownership (1582/1641, 96.4% vs 3092/3529, 87.6%), health or wellness app use (1103/1641, 67.2% vs 1441/3529, 40.8%), and online health information seeking (1367/1641, 83.3% vs 2295/3529, 65%).

Fifth, the following sentence was revised:

Satisfaction with home internet for health needs was also higher in the video group (1075/1641, 65.5% extremely or very satisfied vs 1789/3529, 50.7% among nonusers).

The sentence now reads:

Satisfaction with home internet for health needs was also higher in the video group (1074/1641, 65.4% extremely or very satisfied vs 1790/3529, 50.7% among nonusers).

Finally, the following sentence was revised:

Geographic composition differed across modalities, with video visits more common among residents of large central metros (684/1641, 41.7%) and large fringe metro or suburban areas (393/1641, 23.9%) compared with nonusers (1220/3529, 34.6% and 769/3529, 21.8%, respectively).

The sentence now reads:

Geographic composition differed across modalities, with video visits more common among residents of large central metros (685/1641, 41.7%) and large fringe metro or suburban areas (393/1641, 23.9%) compared with nonusers (1221/3529, 34.6% and 769/3529, 21.8%, respectively).

The correction will appear in the online version of the paper on the JMIR Publications website, together with the publication of this correction notice. Because this was made after submission to PubMed, PubMed Central, and other full-text repositories, the corrected article has also been resubmitted to those repositories.
