# Supplementary material for: Telehealth Use and Modality Choice Among US Adults: Shorrocks-Shapley Decomposition of a 2022 Cross-Sectional National Survey
Source: J Med Internet Res. 2026 Mar 18;28:e81879. doi: 10.2196/81879 (PMC12998708; doi:10.2196/81879)
Supplement: Multimedia Appendix 1 [file jmir-v28-e81879-s001.pdf]

Supplementary Materials

Telehealth Use and Modality Choice Among US Adults in 2022: Cross-Sectional Survey Study Using HINTS 6

Contents

1 Appendix A: Definition of Variables **2**

2 Appendix B: Complete Linear Probability Model Results **4**

2.1 Table B.1: Video vs Audio-Only (Among Telehealth Users) 4
2.2 Table B.2: Video vs No Telehealth 6
2.3 Table B.3: Audio-Only vs No Telehealth 8
2.4 Table B.4: Any Telehealth vs No Telehealth 10

3 Appendix C: Multiple Imputation Sensitivity Analysis **12**

3.1 Little's MCAR Test 12
3.2 Supplementary Missingness Analysis 12
3.3 Imputation Procedure 12
3.4 Comparison of Results 13

4 Appendix D: Supplementary Data Files **14**

4.1 Table S2: Multiple Imputation Regression Results with 95% CIs 14
4.2 Table S3: MI vs Complete-Case Comparison 14
4.3 Table S4: Full MI Regression Results 14
4.4 Little's MCAR Test Output 14
4.5 Table S5: Complete LPM Results – Video vs Audio-Only 14
4.6 Table S6: Complete LPM Results – Video vs No Telehealth 15
4.7 Table S7: Complete LPM Results – Audio-Only vs No Telehealth 15
4.8 Table S8: Complete LPM Results – Any Telehealth vs No Telehealth 15

5 Appendix E: STROBE Checklist **16**

1 Appendix A: Definition of Variables

Table 1: Definition and Coding of Explanatory Variables

Variable	Description & Coding
Sociodemographic Characteristics	
Age	Continuous variable and its quadratic term (Age^2) to capture non-linearities.
Sex	Binary: 1 = Male; 0 = Female.
Marital Status	Binary: 1 = Married or living with a partner.
Race/Ethnicity	Categorical (5 levels): Non-Hispanic (NH) White (Reference), NH Black, Hispanic, NH Asian, NH Other/Multiple Races.
Socioeconomic Status (SES)	
Household Income	Categorical (3 levels): Low ($< \$20,000$, Reference), Middle (\$20,000–$< \$75,000$), High ($\geq \$75,000$).
Education	Binary: 1 = At least a high-school diploma or equivalent.
Employment Status	Categorical indicators: Currently employed (Reference), retired, homemaker, student, other.
Insurance Status	Binary: 1 = Currently has health insurance. Captures all forms of coverage (employer-sponsored insurance, directly purchased plans, government programs such as Medicare and Medicaid, or any other plan that provides medical care or helps pay medical bills).
Health Status & Needs	
Self-Rated Health	Binary: 1 = Excellent or Very Good.
Disability	Binary: 1 = Unable to work due to disability.
Chronic Conditions	Binary indicators: 1 = Ever diagnosed with cancer; 1 = Ever diagnosed with high blood pressure.
Psychosocial Factors	
Social Isolation	Continuous: PROMIS Social Isolation t-score (higher score = more isolated).
Meaning & Purpose	Continuous: PROMIS Meaning and Purpose t-score.
Digital Access & Health Literacy	
Internet Satisfaction	Categorical: Satisfaction with home internet for health needs.
Device Ownership	Binary indicators for owning a smartphone, tablet, etc.
Health Literacy	Categorical: Self-reported ease of understanding medical statistics.
Digital Health Seeking	Binary indicators for using health/wellness apps and seeking health information online.
Geographic Characteristics	
U.S. Census Division	Categorical (9 levels) with Northeast as the reference level.[a]
Urban/Rural Status	Binary: Urban vs. Rural residence, based on the 2013 NCHS classification scheme.[b]

[a] The U.S. Census Bureau defines nine official divisions: **Northeast** (New England, Mid-Atlantic); **Midwest** (East North Central, West North Central); **South** (South Atlantic, East South Central, West South Central); **West** (Mountain, Pacific). [b] NCHS: National Center for Health Statistics.

2 Appendix B: Complete Linear Probability Model Results

The following tables present full coefficient estimates and 95% confidence intervals for all four survey-weighted linear probability models. All models controlled for county-level characteristics (total population, poverty rate, unemployment rate, percent without a high school diploma, and percent of population aged $\geq$65). Standard errors were computed using jackknife replicate weights (50 replicates).

2.1 Table B.1: Video vs Audio-Only (Among Telehealth Users)

Table 2: Survey-weighted LPM: Video vs Audio-Only

VARIABLES	Coefficient (95% CI)
Male	-0.002 (-0.055, 0.052)
Married	0.004 (-0.046, 0.054)
White	0.024 (-0.034, 0.082)
Age 35-49	0.007 (-0.049, 0.062)
Income ≥$75K	0.014 (-0.046, 0.073)
Education (some college+)	0.013 (-0.041, 0.068)
Employed	0.091* (0.026, 0.155)
Disability	0.096* (0.016, 0.176)
Self-Rated Health (Excellent/VG)	0.012 (-0.038, 0.062)
Cancer History	0.023 (-0.040, 0.085)
High Blood Pressure	-0.017 (-0.078, 0.043)
Health Insurance	0.212* (0.130, 0.293)
Census Division (ref: New England)	
Middle Atlantic	0.056 (-0.117, 0.230)
East North Central	0.079 (-0.073, 0.230)
West North Central	0.176 (-0.010, 0.362)
South Atlantic	0.140 (-0.005, 0.286)
East South Central	-0.045 (-0.277, 0.186)
West South Central	0.163 (-0.001, 0.328)
Mountain	0.116 (-0.058, 0.290)
Pacific	0.051 (-0.137, 0.239)
Urbanicity (ref: Rural)	
Large central metro	-0.028 (-0.180, 0.123)
Large fringe metro	0.023 (-0.124, 0.170)
Medium/Small metro	-0.087 (-0.207, 0.033)
Micropolitan	-0.017 (-0.128, 0.093)
Psychosocial	
Isolation (PROMIS T-score)	-0.001 (-0.005, 0.002)
Meaning (PROMIS T-score)	-0.003* (-0.005, 0.000)
Digital Access	
Smartphone only	0.018 (-0.102, 0.137)
Basic cell only	-0.201* (-0.335, -0.068)
Multiple devices	0.055 (-0.055, 0.165)
Internet: Extremely satisfied	0.086* (0.013, 0.159)
Internet: Very satisfied	0.089* (0.009, 0.169)
Internet: Somewhat satisfied	0.011 (-0.072, 0.095)
Health Literacy & eHealth	
Health numeracy: Very easy	-0.033 (-0.142, 0.077)
Health numeracy: Easy	-0.001 (-0.092, 0.090)
Health numeracy: Hard	-0.024 (-0.124, 0.075)
Used health apps	0.055 (-0.034, 0.145)
Did not use apps	0.019 (-0.071, 0.109)
Online health info seeking	0.066 (-0.055, 0.187)
Observations	2,069
R-squared	0.115

* $P < .05$; exact P values available in supplementary CSV files.

2.2 Table B.2: Video vs No Telehealth

Table 3: Survey-weighted LPM: Video vs No Telehealth

VARIABLES	Coefficient (95% CI)
Male	-0.085* (-0.124, -0.046)
Married	0.034* (0.002, 0.066)
White	0.064* (0.023, 0.106)
Age 35-49	0.065* (0.029, 0.101)
Income ≥$75K	-0.011 (-0.051, 0.030)
Education (some college+)	0.002 (-0.035, 0.038)
Employed	0.038 (-0.016, 0.092)
Disability	0.237* (0.178, 0.296)
Self-Rated Health (Excellent/VG)	-0.028 (-0.057, 0.002)
Cancer History	0.068* (0.019, 0.117)
High Blood Pressure	0.058* (0.018, 0.098)
Health Insurance	0.133* (0.092, 0.173)
Census Division (ref: New England)	
Middle Atlantic	0.024 (-0.086, 0.133)
East North Central	-0.075 (-0.185, 0.035)
West North Central	-0.130* (-0.240, -0.020)
South Atlantic	0.028 (-0.083, 0.139)
East South Central	-0.086 (-0.225, 0.052)
West South Central	0.012 (-0.100, 0.125)
Mountain	0.069 (-0.072, 0.210)
Pacific	0.089 (-0.045, 0.223)
Urbanicity (ref: Large central metro)	
Large fringe metro	0.013 (-0.040, 0.066)
Medium/Small metro	-0.045 (-0.104, 0.015)
Micropolitan	-0.035 (-0.132, 0.063)
Rural	-0.052 (-0.176, 0.072)
Psychosocial	
Isolation (PROMIS T-score)	0.001 (-0.001, 0.004)
Meaning (PROMIS T-score)	-0.002 (-0.005, 0.001)
Digital Access	
Smartphone only	-0.051 (-0.115, 0.014)
Basic cell only	-0.074 (-0.164, 0.017)
Multiple devices	0.003 (-0.065, 0.072)
Internet: Extremely satisfied	0.077* (0.011, 0.144)
Internet: Very satisfied	0.058 (-0.012, 0.128)
Internet: Somewhat satisfied	0.003 (-0.062, 0.067)
Health Literacy & eHealth	
Health numeracy: Very easy	0.041 (-0.062, 0.144)
Health numeracy: Easy	-0.022 (-0.118, 0.074)
Health numeracy: Hard	-0.047 (-0.147, 0.052)
Used health apps	0.175* (0.125, 0.226)
Did not use apps	0.060* (0.022, 0.098)
Online health info seeking	0.072* (0.010, 0.134)
Observations	4,317
R-squared	0.158

* $P < .05$; exact P values available in supplementary CSV files.

2.3 Table B.3: Audio-Only vs No Telehealth

Table 4: Survey-weighted LPM: Audio-Only vs No Telehealth

VARIABLES	Coefficient (95% CI)
Male	-0.055* (-0.081, -0.028)
Married	0.013 (-0.014, 0.039)
White	0.037* (0.007, 0.067)
Age 35-49	0.035 (-0.002, 0.071)
Income ≥$75K	-0.003 (-0.036, 0.031)
Education (some college+)	-0.004 (-0.023, 0.016)
Employed	-0.033 (-0.068, 0.002)
Disability	0.119* (0.051, 0.187)
Self-Rated Health (Excellent/VG)	-0.028 (-0.059, 0.002)
Cancer History	0.026 (-0.017, 0.068)
High Blood Pressure	0.058* (0.026, 0.089)
Health Insurance	0.011 (-0.040, 0.063)
Census Division (ref: New England)	
Middle Atlantic	-0.049 (-0.141, 0.043)
East North Central	-0.123* (-0.209, -0.036)
West North Central	-0.185* (-0.254, -0.115)
South Atlantic	-0.093* (-0.184, -0.003)
East South Central	-0.091 (-0.198, 0.016)
West South Central	-0.117* (-0.205, -0.029)
Mountain	-0.056 (-0.156, 0.043)
Pacific	0.023 (-0.107, 0.154)
Urbanicity (ref: Rural)	
Large central metro	0.020 (-0.052, 0.092)
Large fringe metro	0.019 (-0.052, 0.089)
Medium/Small metro	0.033 (-0.016, 0.082)
Micropolitan	-0.012 (-0.063, 0.039)
Psychosocial	
Isolation (PROMIS T-score)	0.001 (-0.001, 0.003)
Meaning (PROMIS T-score)	0.001 (-0.001, 0.002)
Digital Access	
Smartphone only	-0.027 (-0.072, 0.018)
Basic cell only	0.026 (-0.039, 0.091)
Multiple devices	-0.022 (-0.072, 0.029)
Internet: Extremely satisfied	0.001 (-0.054, 0.056)
Internet: Very satisfied	-0.015 (-0.053, 0.022)
Internet: Somewhat satisfied	-0.008 (-0.041, 0.025)
Health Literacy & eHealth	
Health numeracy: Very easy	0.069 (-0.009, 0.146)
Health numeracy: Easy	0.015 (-0.060, 0.089)
Health numeracy: Hard	0.006 (-0.072, 0.083)
Used health apps	0.091* (0.055, 0.127)
Did not use apps	0.040* (0.002, 0.078)
Online health info seeking	0.017 (-0.010, 0.044)
Observations	3,632
R-squared	0.071

* $P <.05$; exact P values available in supplementary CSV files.

2.4 Table B.4: Any Telehealth vs No Telehealth

Table 5: Survey-weighted LPM: Any Telehealth vs No Telehealth

VARIABLES	Coefficient (95% CI)
Male	-0.097* (-0.140, -0.054)
Married	0.031 (-0.006, 0.068)
White	0.071* (0.022, 0.120)
Age 35-49	0.067* (0.022, 0.112)
Income ≥$75K	-0.006 (-0.044, 0.033)
Education (some college+)	0.000 (-0.029, 0.029)
Employed	0.009 (-0.033, 0.051)
Disability	0.225* (0.161, 0.288)
Self-Rated Health (Excellent/VG)	-0.038 (-0.076, 0.001)
Cancer History	0.061* (0.010, 0.112)
High Blood Pressure	0.080* (0.048, 0.112)
Health Insurance	0.115* (0.046, 0.184)
Census Division (ref: New England)	
Middle Atlantic	-0.004 (-0.120, 0.112)
East North Central	-0.126* (-0.191, -0.062)
West North Central	-0.214* (-0.298, -0.130)
South Atlantic	-0.033 (-0.102, 0.035)
East South Central	-0.119* (-0.224, -0.015)
West South Central	-0.060 (-0.150, 0.031)
Mountain	0.018 (-0.077, 0.113)
Pacific	0.077 (-0.019, 0.173)
Urbanicity (ref: Large central metro)	
Large fringe metro	0.017 (-0.039, 0.073)
Medium/Small metro	-0.021 (-0.098, 0.055)
Micropolitan	-0.033 (-0.134, 0.069)
Rural	-0.036 (-0.146, 0.074)
Psychosocial	
Isolation (PROMIS T-score)	0.002 (0.000, 0.004)
Meaning (PROMIS T-score)	-0.001 (-0.004, 0.002)
Digital Access	
Smartphone only	-0.059 (-0.132, 0.014)
Basic cell only	-0.040 (-0.106, 0.026)
Multiple devices	-0.016 (-0.092, 0.059)
Internet: Extremely satisfied	0.051 (-0.013, 0.115)
Internet: Very satisfied	0.029 (-0.037, 0.096)
Internet: Somewhat satisfied	-0.015 (-0.086, 0.057)
Health Literacy & eHealth	
Health numeracy: Very easy	0.062 (-0.016, 0.139)
Health numeracy: Easy	-0.011 (-0.079, 0.057)
Health numeracy: Hard	-0.033 (-0.108, 0.042)
Used health apps	0.184* (0.120, 0.248)
Did not use apps	0.064 (-0.005, 0.133)
Online health info seeking	0.072* (0.024, 0.120)
Observations	5,009
R-squared	0.138

* $P < .05$; exact P values available in supplementary CSV files.

3 Appendix C: Multiple Imputation Sensitivity Analysis

3.1 Little's MCAR Test

We assessed the missing data mechanism using Little's (1988) test for Missing Completely at Random (MCAR). This test evaluates whether the pattern of missing data depends on the observed values by comparing observed variable means across different missing data patterns. The null hypothesis is that data are MCAR.

Table 6: Little's MCAR Test Results

**Statistic**	**Value**
Chi-square (χ^2)	982.1
Degrees of freedom	530
P	<.001

Conclusion: The null hypothesis of MCAR is rejected (P <.001). Data are NOT missing completely at random.

3.2 Supplementary Missingness Analysis

As a supplementary analysis, we examined whether missingness on income (the variable with highest missingness at 11.7%) was predicted by fully observed covariates using logistic regression.

Table 7: Logistic Regression of Income Missingness on Observed Covariates

**Statistic**	**Value**
Likelihood ratio χ^2	1,354.6
Degrees of freedom	18
P	<.001

Conclusion: Missingness is systematically related to observed characteristics (age, census division, cancer history, insurance status). Together with Little's test, these results indicate data are Missing at Random (MAR) conditional on observed variables. Multiple imputation under the MAR assumption is therefore appropriate and will produce unbiased estimates.

3.3 Imputation Procedure

We implemented Multiple Imputation by Chained Equations (MICE) with the following specifications:

- Number of imputations: $m = 20$
- Burn-in iterations: 10
- Imputation models: Logistic regression for binary variables; linear regression for continuous variables
- Variables imputed: Income, race, employment, disability, self-rated health, PROMIS Isolation, PROMIS Meaning, gender, marital status, education
- Auxiliary variables: Age group, census division, urbanicity, cancer history, hypertension, insurance status
- Random seed: 20260115

3.4 Comparison of Results

Multiple imputation estimates were substantively similar to complete-case estimates, supporting the robustness of primary findings. Table S1 presents a comparison of key coefficient estimates between the MI and complete-case analyses.

Table 8: Comparison of MI vs Complete-Case Estimates: Any Telehealth vs No Telehealth

**Variable**	**MI (m=20)**	**Complete Case**
Male	−0.093***	−0.114***
Married	0.052*	0.047*
White	0.048*	0.076**
Age 35-49	0.079**	0.090**
Income ≥$75K	0.040	0.045
Disabled	0.268***	0.235***
Health Insurance	0.168***	0.141**

* P <.05, ** P <.01, *** P <.001; exact P values available in supplementary CSV files.

4 Appendix D: Supplementary Data Files

The following data files are provided as supplementary materials to support transparency and reproducibility:

4.1 Table S2: Multiple Imputation Regression Results with 95% CIs

File: `MI_regression_results_CI.csv`

This file contains the complete regression output from the multiply imputed analysis, including:

- Coefficient estimates (b) for all four contrasts
- 95% confidence intervals (lower and upper bounds)
- P values for each coefficient
- Contrasts: (1) Any telehealth vs none, (2) Video vs no telehealth, (3) Audio vs no telehealth, (4) Video vs audio

4.2 Table S3: MI vs Complete-Case Comparison

File: `MI_vs_CompleteCase_comparison.csv`

This file provides a side-by-side comparison of coefficient estimates from the multiple imputation analysis (m=20) and the complete-case analysis with jackknife variance estimation. This comparison demonstrates the robustness of findings across analytical approaches.

4.3 Table S4: Full MI Regression Results

File: `MI_regression_results.csv`

This file contains the complete set of regression coefficients and standard errors from the multiply imputed analysis across all model specifications.

4.4 Little's MCAR Test Output

File: `littles_mcar_test_results.txt`

This file contains the output from Little's (1988) Missing Completely at Random (MCAR) test, including:

- Variables tested
- Chi-square statistic (χ^2=982.1)
- Degrees of freedom (df=530)
- P <.001
- Conclusion regarding MCAR assumption

4.5 Table S5: Complete LPM Results – Video vs Audio-Only

File: `LPM_VideoVsAudio_Complete.csv`

Complete survey-weighted linear probability model results for the Video vs Audio-Only contrast among telehealth users (N=2,069). Includes all coefficient estimates with 95% confidence intervals. Reference categories: New England (Census Division), Rural (Urbanicity), Tablet only (Device Ownership), Not at all satisfied (Internet Satisfaction), Very hard (Health Numeracy), No smart device (Health app use), No/Not internet user (Health information seeking).

4.6 Table S6: Complete LPM Results – Video vs No Telehealth

File: `LPM_VideoVsNoTelehealth_Complete.csv`

Complete survey-weighted linear probability model results for the Video vs No Telehealth contrast (N=4,317). Includes all coefficient estimates with 95% confidence intervals.

4.7 Table S7: Complete LPM Results – Audio-Only vs No Telehealth

File: `LPM_AudioVsNoTelehealth_Complete.csv`

Complete survey-weighted linear probability model results for the Audio-Only vs No Telehealth contrast (N=3,632). Includes all coefficient estimates with 95% confidence intervals.

4.8 Table S8: Complete LPM Results – Any Telehealth vs No Telehealth

File: `LPM_AnyTelehealthVsNoTelehealth_Complete.csv`

Complete survey-weighted linear probability model results for the Any Telehealth vs No Telehealth contrast (N=5,009). Includes all coefficient estimates with 95% confidence intervals.

Note: All LPM models controlled for county-level characteristics (total population, poverty rate, unemployment rate, percent without a high school diploma, and percent of population aged $\geq$65). Standard errors were computed using jackknife replicate weights (50 replicates). * indicates P <.05.

5 Appendix E: STROBE Checklist

The Strengthening the Reporting of Observational Studies in Epidemiology (STROBE) Statement provides guidance on the reporting of observational studies. The checklist below indicates where each STROBE item for cross-sectional studies is addressed in this manuscript.

Table 9: STROBE Statement—Checklist of Items for Cross-Sectional Studies

Item No.	Recommendation	Reported on Page/Section
**Title and abstract**		
1	(a) Indicate the study's design with a commonly used term in the title or the abstract	Title: "Cross-Sectional Survey Study"; Abstract: "cross-sectional secondary analysis"
	(b) Provide in the abstract an informative and balanced summary of what was done and what was found	Abstract (Background, Objectives, Methods, Results, Conclusions)
**Introduction**		
2	Explain the scientific background and rationale for the investigation being reported	Introduction, paragraphs 1–6
3	State specific objectives, including any prespecified hypotheses	Introduction, final paragraph: "Our objective is to inform policy. . . "
**Methods**		
4	Present key elements of study design early in the paper	Methods: Research Design
5	Describe the setting, locations, and relevant dates, including periods of recruitment, exposure, follow-up, and data collection	Methods: Sampling Procedures—"administered by the National Cancer Institute from February through November 2022"
6	(a) Give the eligibility criteria, and the sources and methods of selection of participants	Methods: Inclusion and Exclusion Criteria; Sampling Procedures
7	Clearly define all outcomes, exposures, predictors, potential confounders, and effect modifiers	Methods: Outcome Variable; Explanatory Variables; Supplementary Materials Appendix A
8	For each variable of interest, give sources of data and details of methods of assessment	Methods: Outcome Variable; Explanatory Variables; Data Linkage; Supplementary Materials Appendix A
9	Describe any efforts to address potential sources of bias	Methods: Survey Design Adjustments (complex survey design, jackknife variance); Missing Data Analysis (MCAR test, MI sensitivity)

Continued on next page

Table 9 – *Continued from previous page*

Item No.	Recommendation	Reported on Page/Section
10	Explain how the study size was arrived at	Methods: Sample Size—"6,252 respondents... adequate statistical power to detect small-to-medium effect sizes"
11	Explain how quantitative variables were handled in the analyses	Methods: Explanatory Variables; Digital Readiness Variables; Supplementary Materials Appendix A
12a	Describe all statistical methods, including those used to control for confounding	Methods: Primary Analyses (survey-weighted LPMs); Decomposition Analysis (Shorrocks-Shapley)
12b	Describe any methods used to examine subgroups and interactions	Methods: Primary Analyses—four binary contrasts (video vs. audio, video vs. none, audio vs. none, any vs. none)
12c	Explain how missing data were addressed	Methods: Missing Data Analysis; Multiple imputation (MICE, m=20); Supplementary Materials Appendix C
12d	If applicable, describe analytical methods taking account of sampling strategy	Methods: Survey Design Adjustments—"jackknife replicate weights... design-consistent point estimates and robust standard errors"
12e	Describe any sensitivity analyses	Methods: Missing Data Analysis—MI sensitivity analysis; Supplementary Materials Appendix C
**Results**		
13a	Report numbers of individuals at each stage of study	Results: Sample Characteristics—"Among 6,252 respondents... n=1,641 reported any video telehealth, n=876... audio-only... n=3,529... no telehealth"
13b	Give reasons for non-participation at each stage	Methods: Inclusion and Exclusion Criteria (HINTS sampling frame exclusions); Not applicable for secondary data analysis
13c	Consider use of a flow diagram	Not included (secondary analysis of existing survey data)
14a	Give characteristics of study participants	Results: Sample Characteristics by Telehealth Modality; Table 1
14b	Indicate number of participants with missing data for each variable of interest	Results: Missing Data Patterns—missingness rates reported for PROMIS scores, employment, disability, race/ethnicity, income

Continued on next page

Table 9 – *Continued from previous page*

Item No.	Recommendation	Reported on Page/Section
15	Report numbers of outcome events or summary measures	Results: Sample Characteristics—survey-weighted prevalence: video 27.2%, audio-only 12.1%, none 60.7%
16a	Give unadjusted estimates and, if applicable, confounder-adjusted estimates and their precision	Results: Regression Estimates; Table 2 (adjusted estimates with 95% CIs); Supplementary Materials Appendix B (full results)
16b	Report category boundaries when continuous variables were categorized	Supplementary Materials Appendix A (income: <\$20K, \$20K–<\$75K, ≥\$75K; age groups)
16c	If relevant, consider translating estimates of relative risk into absolute risk	Results: coefficients reported as percentage-point (pp) differences (absolute risk differences)
**Other analyses**		
17	Report other analyses done—eg analyses of subgroups and interactions, and sensitivity analyses	Results: Shorrocks-Shapley Decomposition; Missing Data Patterns and Multiple Imputation Sensitivity Analysis
**Discussion**		
18	Summarise key results with reference to study objectives	Discussion, paragraphs 1–3: "we find that who uses telehealth and how they use it are shaped by distinct factors…"
19	Discuss limitations of the study, taking into account sources of potential bias or imprecision	Discussion: Limitations—cross-sectional design, self-reported measures, county-level broadband limitations, modest R^2
20	Give a cautious overall interpretation of results considering objectives, limitations, multiplicity of analyses, results from similar studies, and other relevant evidence	Discussion: Policy and Practice Implications; comparison to prior literature
21	Discuss the generalisability (external validity) of the study results	Discussion: Limitations—"survey-weighted national scope…comprehensive, policy-relevant portrait of telehealth determinants in 2022"
**Other information**		
22	Give the source of funding and the role of the funders for the present study	Funder Role Statement: "Health Resources and Services Administration. Grant number: U3GRH40001"

Reference: von Elm E, Altman DG, Egger M, Pocock SJ, Gøtzsche PC, Vandenbroucke JP;

STROBE Initiative. The Strengthening the Reporting of Observational Studies in Epidemiology (STROBE) statement: guidelines for reporting observational studies. *Lancet.* 2007;370(9596):1453–1457.
